# Brain-on-a-Chip: Characterizing the next generation of advanced *in vitro* platforms for modeling the central nervous system

**DOI:** 10.1063/5.0055812

**Published:** 2021-07-30

**Authors:** Ben M. Maoz

**Affiliations:** 1 Department of Biomedical Engineering, Tel Aviv University, Tel Aviv 69978, Israel; 2 Sagol School of Neuroscience, Tel Aviv University, Tel Aviv 69978, Israel; 3 The Center for Nanoscience and Nanotechnology, Tel Aviv University, Tel Aviv 69978, Israel

## Abstract

The complexity of the human brain creates significant, almost insurmountable challenges for neurological drug development. Advanced *in vitro* platforms are increasingly enabling researchers to overcome these challenges, by mimicking key features of the brain's composition and functionality. Many of these platforms are called “Brains-on-a-Chip”—a term that was originally used to refer to microfluidics-based systems containing miniature engineered tissues, but that has since expanded to describe a vast range of *in vitro* central nervous system (CNS) modeling approaches. This Perspective seeks to refine the definition of a Brain-on-a-Chip for the next generation of *in vitro* platforms, identifying criteria that determine which systems should qualify. These criteria reflect the extent to which a given platform overcomes the challenges unique to *in vitro* CNS modeling (e.g., recapitulation of the brain's microenvironment; inclusion of critical subunits, such as the blood–brain barrier) and thereby provides meaningful added value over conventional cell culture systems. The paper further outlines practical considerations for the development and implementation of Brain-on-a-Chip platforms and concludes with a vision for where these technologies may be heading.

## INTRODUCTION

Neurological disorders take a vast societal toll, claiming some 9 million lives each year,^1^ and imposing an annual economic burden exceeding $700 billion in the U.S. alone.^2^ Current estimates suggest that, in the U.S., more than 7 million adults suffer from neurodegenerative diseases (e.g., Alzheimer's disease—5 million; Parkinson's disease—1 million; multiple sclerosis—400k), and at least 15% of children under the age of 17 have been diagnosed with neurodevelopmental diseases.^3^ Overall, neurological disorders are estimated to account for 6.3% of the disease burden worldwide.^4^ Yet, drug development for central nervous system (CNS) disorders remains notoriously failure-prone, with CNS drugs taking 38% longer to be approved compared with non-CNS drugs.^5^ As a result of these difficulties, between the years 2005 and 2014, many leading pharmaceutical companies substantially reduced their CNS drug development programs and even eliminated them altogether.^6^ This trend is of significant concern to policy makers, who are responding with initiatives to promote innovation in CNS research, toward overcoming the extreme challenges associated with this domain (see below for a discussion of some of these challenges).

The primary pathway to innovation in CNS research is through the development of novel *in vitro* CNS models. In general, *in vitro* technologies have made enormous strides in recent years and are becoming so advanced that they may soon replace animal models in many applications^7^ (see Refs. 8–12 for recent reviews of the use of novel *in vitro* tools in CNS studies). Prominent advanced *in vitro* platforms include, among others, 3D-printed models,^13^ organoids,^14^ and Organs-on-a-Chip (OoCs).^15^ The latter, also referred to as microphysiological systems, are based on microfluidic chips containing miniature engineered tissues that represent specific organs and, ideally, recapitulate their key functions. It is predicted that adoption of OoC systems in commercial and academic labs may eventually reduce research and development costs for each new drug by 10%–26%, within a timescale of 5 years.^16^ Accordingly, OoCs hold obvious appeal for CNS research, and several microfluidics-based CNS modeling systems (i.e., “Brains-on-a-Chip”) have already been developed—as discussed in numerous perspectives^17–21^ and reviews.^12,22–35^

In light of the promise embedded in microfluidic OoC platforms, the phrase “Brain-on-a-Chip” (BoC) has become highly popular as a term to describe diverse *in vitro* modeling systems targeting the CNS—systems that do not necessarily rely on microfluidics. This Perspective aims to refine the definition of the term Brain-on-a-Chip for future *in vitro* technologies, to ensure that it continues to signify innovation and meaningful added value over conventional cell culture systems. To this end, the following sections (i) elaborate on the expectations for a Brain-on-a-Chip system—and, more specifically, on the challenges that such a system should aim to overcome; (ii) discuss, in broad terms, how microfluidic OoC systems can overcome some of these challenges; (iii) identify specific criteria for what systems should qualify as a Brain-on-a-Chip (based on the extent to which the system addresses the unique challenges associated with CNS modeling—regardless of whether it is based on microfluidics technology); (iii) provide a brief overview of the capabilities of current Brain-on-a-Chip systems; (iv) outline some of the practical considerations associated with the development or implementation of Brain-on-a-Chip platforms; and (v) present a broad vision for where Brain-on-a-Chip technology is heading.

## THE CHALLENGES OF MODELING THE BRAIN (CNS) *IN VITRO*

**FIG. 1.**
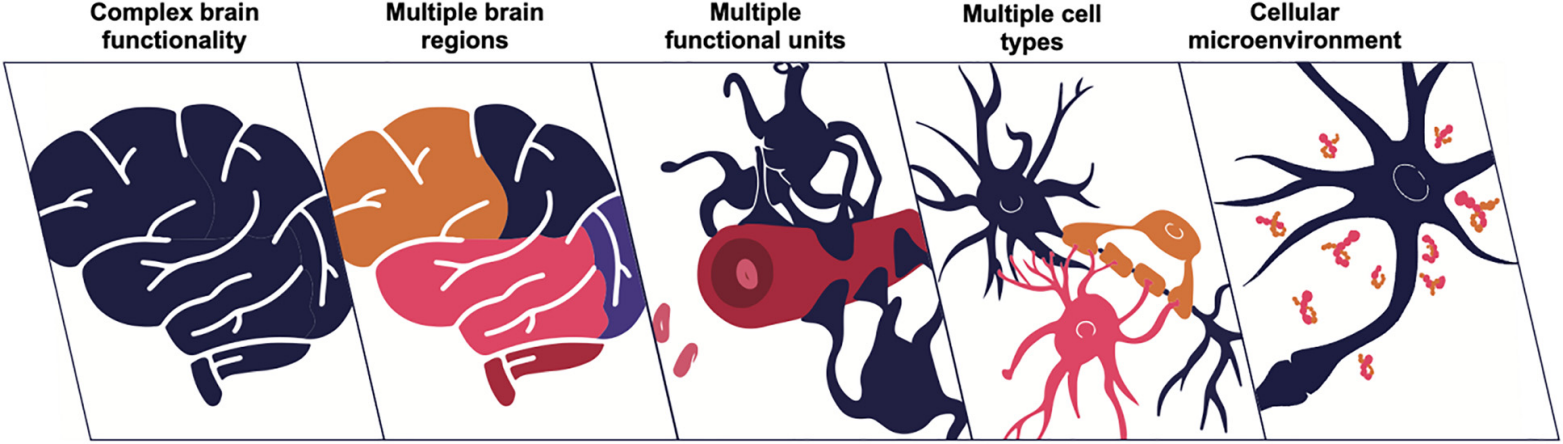
The brain is a multiscale system.

**FIG. 2.**
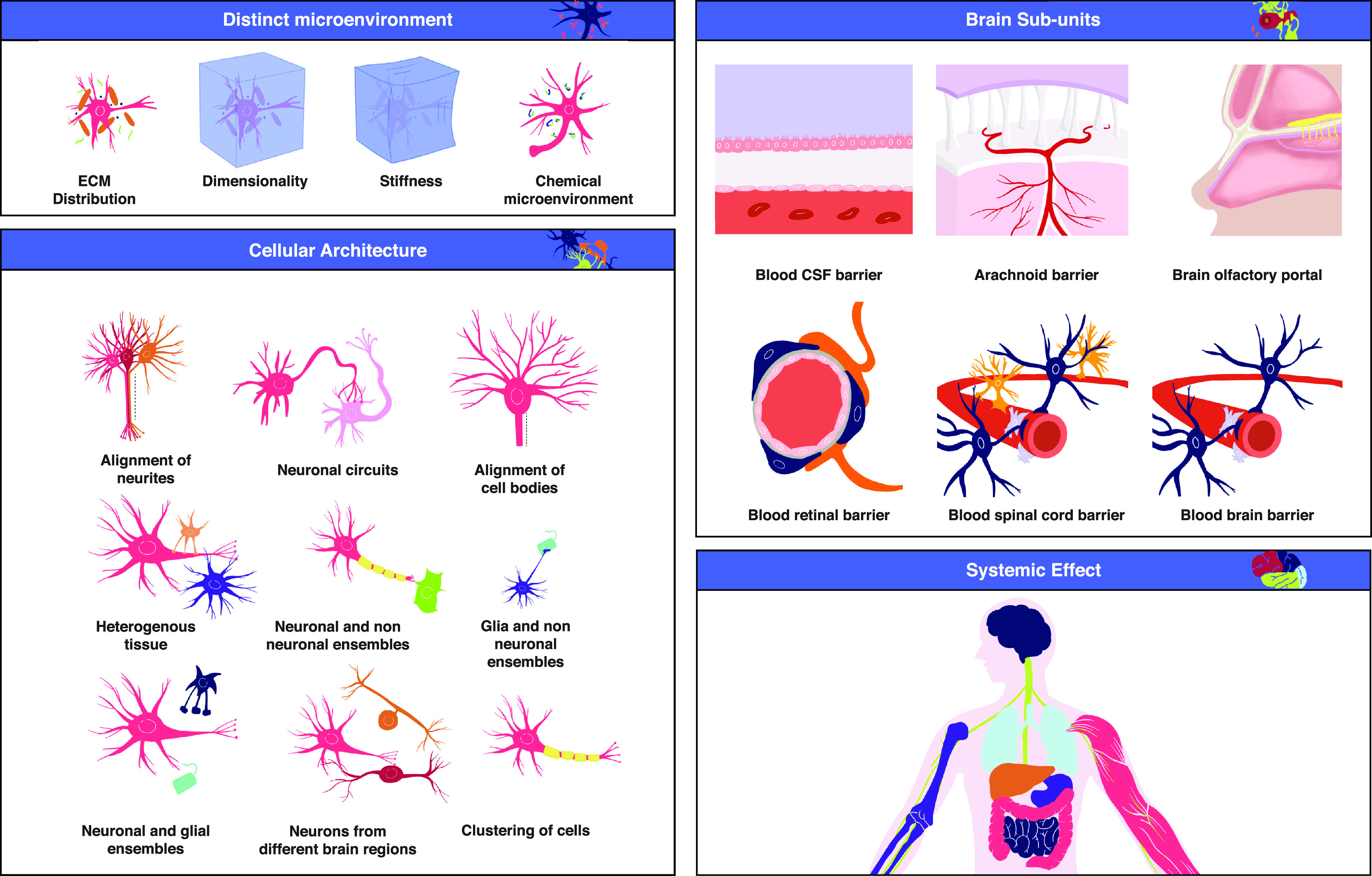
Different aspects of the brain's complexity. As the brain is a multiscale system, it is challenging to incorporate all these aspects *in vitro*. One should keep in mind all these aspects when selecting the appropriate *in vitro* platform for the specific problem one is interested in studying. Once the platform has been selected, the researcher can use Table I to determine whether the platform qualifies as a Brain-on-a-Chip, i.e., whether its capabilities exceed those of conventional cell culture systems.

Before discussing how a brain might be recapitulated “on a chip,” it is important to clarify the expectations for an *in vitro* system aimed at modeling the brain, and the challenges that such a system must strive to overcome. The brain is a multiscale system (Fig. 1), and its complexity makes the challenges associated with *in vitro* modeling particularly severe. Indeed, these challenges are the key factors underlying the substantial failure rate of CNS drug development efforts (Fig. 2).

### Challenge 1: The brain's distinct microenvironment

The brain's microenvironment is significantly different from the microenvironments characterizing other organs. One key feature of the brain's microenvironment is the substantial proportion of extracellular space—occupying ∼20% of brain tissue. Moreover, in the brain, small molecules have an effective diffusion coefficient that is two-fifths that in free solution. Recent studies suggest that this microenvironment has a major role in brain homeostasis both in health^36^ and in disease, and changes in the microenvironment have been linked to the onset of various neurodegenerative diseases.^37–39^

The challenge of reproducing the brain's microenvironment *in vitro* includes multiple aspects,^25,40–42^ and currently, no single *in vitro* platform can overcome all of them:

(1) *Incomplete characterization of the brain microenvironment, in general, and of the extracellular matrix (ECM), in particular*: Though extensive research efforts have recently been devoted to characterizing the brain microenvironment, critical gaps remain. In particular, the ECM—which is arguably one of the most important components of the brain microenvironment, given its crucial role in cell growth, mechanotransduction, and cell signaling,^25,41^—has only been partially characterized and mostly in the rodent brain.^43^
(2) *Unique composition of the microenvironment*: The brain's microenvironment contains unique proteins and other molecules that are not abundant in the rest of the body. Examples of such molecules include glycosaminoglycans (GAGs), such as hyaluronic acid (HA); proteoglycans, such as brevican, neurocan, and phosphacan; and others. Though *in vivo* neurons are surrounded by these proteins and molecules, it is extremely challenging to integrate them into neuron cultures *in vitro.*^8,25,41^ In addition, these proteins and molecules are not easy to extract, which makes them very expensive relative to common proteins, such as collagen, poly-lysine and others, and less common in standard *in vitro* models.^25,41^ Even if these challenges were to be overcome, it would not necessarily be possible to derive a straightforward representation of the brain microenvironment, as recent studies show that the various proteins and other molecules are not equally distributed across brain regions; rather, each brain region has a unique ECM composition.^43^
(3) *Mechanical properties*: In recapitulating the brain's mechanical properties, it is necessary to take two important parameters into account. The first is *stiffness*: the brain is considered to be one of the softest tissues in the body (∼50 Pa). However, the most common *in vitro* culture methods use plastic and glass substrates, which provide high mechanical stiffness (GPa), orders of magnitude higher than that of the *in vivo* environment—a substantial limitation, given that substrate stiffness is known to have a significant influence on cell behavior, differentiation, and migration, and in the shaping of *in vitro* cell phenotypes.^25^ The second parameter is *viscoelasticity*, that is, the brain's known capacity to dissipate stress over time when strain is applied.^44^ Thus far, no single brain cell culture platform has simultaneously recapitulated the brain's physiological stiffness and viscoelasticity. Consequently, brain tissue cells cultured *in vitro* often display different morphology and functions compared with *in vivo* cells.^45^
(4) *Dimensionality*: Clearly, the brain is a 3D structure, whereas most standard *in vitro* models are 2D. Dimensionality has a significant role in the cellular microenvironment, as it determines how cells can move, the distribution of forces applied on the tissue, and the directions from which physiochemical signals can arrive. Thus, reliance on a 2D platform constitutes a significant simplification of the *in vivo* brain, limiting the dynamics of the neuronal network and hindering integration with other cells. Nevertheless, 2D models have great advantages, as they are easy to work with, and they absorb significantly less material from the medium compared with 3D models. It is important to note that the term “3D” does not refer only to the 3D environment of the cells; it can also refer to the 3D structure of the tissue (e.g., dispersed cells in 3D gel vs. 3D organoids).
(5) *Chemical microenvironment*: The human brain has a unique chemical microenvironment, which includes specific oxygen levels, multiple fluid types with specific nutrient compositions [i.e., blood on the vasculature side and cerebrospinal fluid (CSF) on the parenchymal side], and specific growth factors that are secreted from other cells. It is difficult to mimic these exact conditions *in vitro*; for example, standard *in vitro* setups rarely incorporate controls over oxygen levels, and the resulting differences between the *in vitro* and *in vivo* environments can influence cell metabolism.^46^ More broadly, non-physiological culture conditions—some of which may be explicitly designed to optimize various outcomes, such as rapid cell growth—can induce genetic and phenotypic changes.^47^

### Challenge 2: Cellular architecture

The connections among brain cells create a unique cellular architecture. These connections are dynamic and change constantly, in what is known as neural plasticity. The brain's functionality is highly dependent on the cellular architecture—the placement of the axons, the types and numbers of cells they interact with, and the “strength” of the synaptic connections; furthermore, it is known that changes in neuronal connections can indicate on the onset of disease or neuronal degradation. Researchers have long recognized the significance of the brain's cellular architecture and sought to elucidate it; indeed, in 2010, a vast NIH-sponsored initiative called the Human Connectome Project was established, with the goal of mapping the connections within the brain.^48^

### Challenge 3: Multiple cell types

The brain contains about 100 billion neurons and up to ten times more glia cells.^49^ In addition to neurons and astrocytes, the brain contains endothelial cells, pericytes, microglia, oligodendrocytes, immune cells, and more. Each of these classes of cells may comprise subtypes of cells—for example, the neurons alone include more than 500 subtypes, and the full extent is still unknown.^50^ The fact that there are so many cell types, with each one interacting in its own unique way with each of the other cell types, makes it almost impossible to capture all types of cell–cell interactions *in vitro*. Examples of important interactions include the following: astrocytes provide metabolites for neurons; oligodendrocytes myelinate neurons; pericytes affect the permeability of the endothelium (blood–brain barrier; BBB); and microglia trigger astrocytic reactions.

### Challenge 4: Different brain regions with unique functionalities

The phrase Brain-on-a-Chip implies a single system capturing the brain as a single organ. Yet, the brain is composed of more than 250 different brain regions,^51^ each of which is characterized by a unique microenvironment, cellular composition, architecture, connectivity, and most importantly, functionality. Moreover, it is known that some brain regions are more prone to certain neurological diseases and disorders than others.^52–54^ It is important to keep these points in mind when constructing an *in vitro* model of “the brain,” particularly for drug development purposes. At the same time, several challenges hinder the development of *in vitro* models that incorporate more than one region—including a lack of reliable, affordable, and reproducible cells sourced from different human brain regions; the lack of characterization of the unique composition of each brain region; and the need to connect the different regions in order to create a multifunctional unit.

### Challenge 5: Multiple subunits

The brain contains several unique physiological systems that are crucial to its functionality, both in homeostasis and in disease, and that have a significant role in drug delivery. These systems include the brain endocrine system; the choroid plexus, which produces CSF; the glymphatic system, which is responsible, among other things, for removal of waste from the CSF; and the vasculature system and its barriers (BBB, blood–CSF barrier, blood–retinal barrier, blood–spinal cord barrier, and the arachnoid barrier), which control the influx and efflux of nutrients. It is essential to integrate these systems *in vitro*, especially when one is interested in toxicology and drug assessment. Unfortunately, it is very challenging to reproduce the BBB *in vitro* in a manner that recapitulates its *in vivo* barrier properties.^55–58^ It is similarly challenging to recapitulate the other units, not least because it is difficult to obtain human-relevant cells from these units (e.g., the choroid plexus) that can be incorporated into *in vitro* platforms.

### Challenge 6: Systemic effect—Organ–organ interactions

The brain is part of a complex physiological system and does not operate in a “vacuum.” Indeed, this statement is true for most organs, not just the brain—yet, standard *in vitro* models do not capture organ–organ interactions. An absence of organ–organ interaction severely limits the capacity to study the brain in its role as the regulator of all bodily functions as well as to understand the feedback loops of hormones released by the endocrine system, which drive much of the brain's functionality.

### Challenge 7: Brain functionality

Like many other organs, the brain does not have just one defined functionality. However, whereas the functions of many other bodily organs can be replaced or compensated for, e.g., through chemical or mechanical means, the brain's functionalities are impossible to fully mimic with today's tools. The brain is responsible for monitoring the body's homeostasis, processing sensory inputs, and controlling the output. Ultimately, the brain makes us who we are, through cognition, self-awareness, and consciousness. It is currently impossible to capture all these aspects of the brain's functionality in an *in vitro* system. Thus, most models suffice with a simplified definition of the brain's “functionality:” Specifically, they target the neurons, the basic building block of the brain, and evaluate their functionality by measuring their electrophysical activity. It is currently impossible to understand how changes in neuronal electrical activity or connectivity might translate into changes in higher-order functions, such as self-awareness or consciousness.

## USING OOC PLATFORMS TO OVERCOME CNS MODELING CHALLENGES

The concept of an OoC was first realized in 2010, when Huh *et al.* developed a microfluidic chip that reproduced the human alveolar–capillary interface, successfully replicating the physiological functionality of a breathing lung.^59^ Since then, numerous OoC systems have been developed, starting from single organs (e.g., heart, brain, liver, kidney, etc.) and up to systems of multiple organs,^60,61^ composed of as many as eight linked OoCs, constituting a “Mini Body on a Chip.”^62^

Broadly, an OoC consists of one or more cell cultures in a microfluidic chip, in which it is possible to recapitulate vascular perfusion, tissue–tissue interfaces, and organ‐relevant mechanical motions, while also allowing integration of circulating immune cells, connective tissue cells, and a complex microbiome. Moreover, these systems allow for application of physical, mechanical, and chemical stimuli, to mimic the human microenvironment. OoCs provide the capacity to identify new disease mechanisms and new physiology, and to correlate *in vitro* data with clinical studies^63^ (see reviews in Refs. 7, 15, and 64–67, in addition to two excellent stakeholder workshops,^68,69^ for overviews of the field).

Many of these features provide direct benefits in CNS modeling that are not attainable through conventional *in vitro* platforms. For example, a primary advantage of OoC systems is the capacity to incorporate flow. This feature enables shear forces to be applied to the cells, enables nutrients and drugs to perfuse at different concentrations and timepoints, and enables different OoCs to be connected to each other, thereby providing the capacity to mimic organ–organ interactions—overcoming some of the key CNS modeling challenges outlined above (challenges 1, 4, 5, and 6). Such capabilities are further enhanced by chip designs that enable different cell types to be co-cultured, either on top of a membrane, in 3D gels, compartments, and more, thereby creating cell–cell interactions resembling the *in vivo* microenvironment. The absence of flow from conventional, static cultures not only precludes these capabilities but can also lead to a buildup of nutrients in the dish or well, which can influence cellular functionality and growth. In light of these advantages, numerous microfluidics-based Brain-on-a-Chip platforms have been developed in recent years (see the “State of the Art” section for further discussion of these platforms).

## CRITERIA FOR A NEXT-GENERATION BRAIN-ON-A-CHIP

Though microfluidic OoCs provide significant opportunities for overcoming the challenges of CNS modeling, not every system that is referred to as a Brain-on-a-Chip relies on microfluidic technology (see the State of the Art section for further discussion). Indeed, as noted in the introduction, researchers and practitioners have adopted the term Brain-on-a-Chip to refer to a vast array of advanced *in vitro* systems targeting the CNS. The basic objective of this paper is to refine the definition of the term Brain-on-a-Chip for future generations of *in vitro* platforms, to ensure that the concept continues to refer to systems that provide meaningful advantages over conventional *in vitro* approaches.

More specifically, this paper suggests that to provide such meaningful advantages, a Brain-on-a-Chip platform must address some of the unique CNS modeling challenges outlined above—to an extent that exceeds the capacities of conventional *in vitro* techniques. For example, some Brain-on-a-Chip platforms, unlike conventional cell cultures, enable a functional BBB layer to be integrated into cultures of brain parenchymal cells (thereby addressing Challenge 5). Such platforms include (but are certainly not limited to) microfluidics-based platforms, which provide an opportunity to induce shear stress on the BBB layer, significantly enhancing the BBB properties, compared with static models.^70–72^ The BBB serves as the brain's gatekeeper and is one of the limiting factors in delivering drugs to the brain. Accordingly, inclusion of a BBB in an *in vitro* model can provide critical insights for drug development, circumventing the inability of conventional models to predict drug effects.

Table I presents a back-of-the-envelope approach for quantifying the added value of an *in vitro* model over conventional systems and determining whether a particular platform should qualify as a Brain-on-a-Chip. Specifically, in the table, each of the potential features of an *in vitro* system—classified according to their capacity to address the CNS modeling challenges elaborated above—is assigned a numerical score. I suggest that a platform that achieves a cumulative score of 2 or above can be defined as a Brain-on-a-Chip. As *in vitro* technologies continue to develop, and more advanced platforms become the standard, the threshold for qualifying as a Brain-on-a-Chip may increase.

**TABLE I.** Criteria for quantifying the added value of an *in vitro* system over conventional cell culture systems and for determining whether the system should qualify as a Brain-on-a-Chip. (A platform that achieves a total score of at least 2 can qualify as a Brain-on-a-Chip.)

Property	Example of different features	Comments
Challenge 1: mimics the brain's microenvironment	Stiffness, native ECM, mechanical forces	
• Mimicking the brain's native ECM (1)		
• Inducing mechanical forces (1)		
• Mimicking the 3D structure (1)		
• Mimicking cellular connectivity (1)		
• Creating an *in vivo* like physiochemical microenvironment (1)		
Challenge 2: recapitulates cellular architecture (2)	Substrates that induce desired network properties or mimic a physiological architecture	
Challenge 3: integrates different cell types (1)	Incorporates not only neurons and astrocytes but also brain endothelial microvasculature, pericytes, microglia, and oligodendrocytes	It is imperative to demonstrate proper cell functionality in the platform—including, e.g., cellular architecture and connectivity. Just co-culturing cells together is not sufficient, as this can be done with conventional tools.
Challenge 4: integrates multiple brain regions (3)	Connects between the prefrontal cortex, amygdala, and hippocampus	This can include the cellular architecture and connectivity. Connections can be either physical or chemical or both.
Challenge 5: integrates brain subunits (3)	Choroid plexus, glymphatic system, BBB, blood–CSF barrier, blood–retinal barrier, blood–spinal cord barrier, and the arachnoid barrier	
Challenge 6: systemic effect—organ–organ interactions (4)	Linking the brain to other organs	
Challenge 7: mimicking advanced brain functionality (5)		Currently there are no “Platforms-on-a-Chip” with this capacity. There are, however, some advanced neuronal *in vitro* models that use neuronal platforms for advanced applications (e.g., controlling flight simulators^73^ and computer software^74^).

## STATE OF THE ART

Numerous *in vitro* platforms, developed in recent years, integrate multiple properties mentioned in Table I and thus effectively fulfill the criteria for being referred to as Brain-on-a-Chip systems. These platforms have been discussed extensively (see Refs. 8–12, 42, and 75 for reviews of advanced *in vitro* models of the CNS, in general; and see Refs. 17–35 and 76 for specific reviews of the Brain-on-a-Chip). Accordingly, in what follows, I will provide only a brief overview of these technologies. As summarized in Table II, different reviews present different approaches for classifying Brain-on-a-Chip systems. For example, systems can be classified according to the dimensions of the model (Table II, lines 1–3); the anatomical system that is being modeled (Table II, cell 4); the cells that are being used (Table II, cell 5); or the anatomical scale (Table II, cell 6). In line with the focus of this Perspective, in the following overview, I categorize Brain-on-a-Chip technologies according to their capacity to address each of the CNS modeling challenges summarized in Table I. I note that, as yet, no Brain-on-a-Chip technologies exist that address Challenge 7, i.e., recapitulation of advanced brain functionalities, such as cognition.

**TABLE II.** Different approaches for categorizing Brain-on-a-Chip (BoC) platforms.

Categories for classifying Brain-on-a-Chip platforms										Ref.
Models categorized by the Brain-on-a-Chip dimensions										
1.	Surface-based designs				Bulk-based designs					26
2.	2D BoC for axon isolation	BoC with porous membrane			3D high-content BoC with hydrogel		Interconnected BoC system	BoC integrated with well plate		19
3.	Spatially patterned 2D	Microfluidic 2D		Compartmentalized 2D or 3D			Hydrogel 3D	Spheroid 3D		23
4.	2D BBB-BoC	2.5D BBB-BoC		3D BBB-BoC			3D NVU-BoC	3D NVU(+)-BoC		76
Anatomical system										
5.	CNS models						Peripheral nervous system (PNS) models			18,34
Cell based										
6.	Neuronal-stem-cell based microfluidic system						Human iPSC-based microfluidic system			
Anatomical scale										
7.	Molecular level		Cellular level			Tissue level			Organism level	27

### Challenge 1: Mimicking the brain's microenvironment

As noted above, it is a great challenge to completely mimic the brain microenvironment,^25,41,42^ and as yet, no single platform integrates all features. Yet, significant advances have been made in recapitulating certain individual characteristics of the microenvironment. Recent reviews nicely summarize new materials that demonstrate a similar *stiffness* to the brain,^25^ models that recapitulate the brain's *3D structure*,^34^ and models that incorporate native ECM materials.^41,77^ One study of note is the work of Lam *et al.*, who demonstrated neuronal and glial co-culture on brain-tissue-specific ECM, including full characterization of the cells' electrical activity.^78^ Another notable advancement relates to the development of 3D neuronal tissues, in a method known as organoids. Organoids are 3D cellular structures that can demonstrate unique 3D architecture, enable integration of multiple cell types, demonstrate different functionalities, and can mimic different brain regions (such as “cerebral organoids,” “forebrain organoids,” or “midbrain organoids”).^79^ Recent models have integrated organoids with unique chip systems, enabling multiple organoids to be linked (see Ref. 80 for a review). These linked systems of “Organoids-on-a-Chip” can accommodate different cell populations and functionalities, and mimic different diseases.^81–84^

### Challenge 2: Cellular architecture

Traditional neuronal cultures are anisotropic, meaning that neurons are cultured without any particular directionality. Consequently, the resultant network architectures do not closely resemble *in vivo* architectures; i.e., they are typically random, without a specific adhesion site, and lack directionality and connectivity.^85^ In recent years, the development of new methods, such as microfabrication, microfluidic chips and microchannels, microcontact printing, 3D printing, and surface treatments, has enabled researchers to create neuronal cultures with controlled patterns and connections. Indeed, the ability to control neuronal directionality and connectivity is now well established in 2D systems,^85–88^ facilitating investigation of the interplay between anatomical connectivity and dynamics in neural networks (albeit in a simplified manner). For example, in a pioneering work, Fienerman *et al.*^89^ created reliable neuronal logic circuits that can act as a diode, an AND gate, and a delay circuit. Subsequent studies created neuronal circuits with defined functionality.^90^ These milestones are laying the groundwork for the development of neuronal computers, which will execute the computational tasks using living neurons. It should be mentioned, though, that despite the advancements achieved in controlling neuronal architecture in 2D, it is still a great challenge to do so in 3D.

### Challenge 3: Integration of different cell types

Though conventional *in vitro* methods can be used to co-culture certain types of brain cells—e.g., astrocytes and neurons—other types of co-cultures are more challenging, requiring the use of more advanced technologies (see Refs. 91–93 for recent examples of the use of novel methods to co-culture and integrate multiple brain cell types, and to validate their interactions). For example, it is highly complex to co-culture neurons with oligodendrocytes that myelinate the neurons. Recently, several groups demonstrated the use of microfluidic chips to achieve co-cultures in which oligodendrocytes myelinated neurons from the CNS^94^ as well as from the PNS;^95^ these studies demonstrated how myelination can affect neuronal functionality (see Ref. 96 for further discussion of the challenges of reproducing neuronal myelination *in vitro*). A similar approach was used to study neuronal–glial interactions,^97,98^ and interactions between neurons and astrocytes; such studies identified, for example, how α-synuclein aggregates propagate in Parkinson's disease^99^ and elucidated the effect of α-synuclein propagation on neurite growth.^100^ Recent *in vitro* models were able to use advanced fabrication methods, such as 3D printing and microfabrication, to integrate multiple cell types (e.g., neurons, astrocytes, glia, endothelial cells) in a specific 3D spatial design and to use them for studying neurodegenerative diseases, such as Alzheimer's disease^101^ and brain cancer.^102^

### Challenge 4: Integration of multiple brain regions

As mentioned above, it is a great challenge to create an *in vitro* model that integrates different brain regions. Therefore, only a few such models exist, and most of them use rodent cells and tissues—which are more accessible than human cell sources—to mimic the different connections: cortex and thalamus,^103^ cortical and striatal,^104^ hippocampal and cortical,^105^ prefrontal cortex, hippocampus and amygdala,^86^ hippocampal and cortical,^106^ thalamic, and hippocampal and cortical.^107^ Ndyabawe *et al.* recently developed a multicompartment platform that enables the integration of different human neuronal stem cells, representing the composition of specific brain regions.^108^ Another recent work created brain region-specific organoids, which were used to model the effect of exposure to the Zika virus.^109^ Despite these recent advancements, there is still much room for development of Brain-on-a-Chip platforms that recapitulate the composition, characteristics, connections, and functionality of the different brain regions.

### Challenge 5: Integration of brain subunits

In recent years, significant developments have been made in mimicking brain subunits on a chip. In particular, much focus has been devoted to the BBB, due to its importance in drug development and toxicology.^110^ Many prior reviews have covered advanced *in vitro* models of the BBB;^55,56,111,112^ of particular interest in our context are models that integrate BBB units with neurons, effectively mimicking the neurovascular unit (NVU). There are two main approaches for creating such models: (i) “all in one,” in which many cellular components are integrated in one platform; and (ii) “linked systems,” comprising multiple chips—each of which mimics a specific functionality—linked together to create a larger, more complex functional unit. The technologies underlying all-in-one systems include Transwells,^93,113^ porous-tube models (with astrocytes),^114^ ECM-based microfluidic chips,^115,116^ and organoids (neuronal core and endothelial shell).^117^ Linked systems, in turn, typically rely on microfluidic OoC technology, which, as discussed above, provides the capacity to link multiple organ-units. Maoz *et al.*, for example, introduced an OoC system in which a BBB-chip was linked to a chip containing brain parenchymal cells; the researchers used this system to identify previously unknown endothelium–neuronal metabolic coupling.^92^ In addition to the BBB, other brain units have been recapitulated in advanced *in vitro* models—including the Retina-on-a-Chip^118–120^ and the Mucosa-on-a-Chip.^121^ Recently, Pellegrini *et al.* successfully created a 3D brain organoid that includes the choroid plexus and is able to produce CSF *in vitro.*^122^

### Challenge 6: Systemic effect—Organ–organ interactions

Recently, several groups have recapitulated interactions between multiple organs (including the brain) by coupling several different OoCs.^60–62,123^ Such multi-OoC systems have been demonstrated in toxicology studies, with results corresponding to published data.^60,61^ Another study successfully modeled physiologically based pharmacokinetics (PBPK) of a marker in a system comprising eight linked OoCs.^62^ Recently, Trapecar *et al.* used a multi-OoC system to demonstrate that systemic interaction between a Gut–Liver-Chip and a Brain-on-a-Chip enhances the features of *in vivo*-like behavior of cerebral micro-physiological system (MPS), and that microbiome-associated short-chain fatty acids increase the expression of pathways associated with Parkinson's disease pathology.^123^ While there are some recent reviews of multi-OoC platforms (for example, Refs. 7, 15, 64–67, and 124–127), this technology is rapidly developing, offering a significant promise, alongside many challenges to overcome.

## PRACTICAL CONSIDERATIONS IN DEVELOPING OR IMPLEMENTING A BRAIN-ON-A-CHIP PLATFORM

A researcher seeking to develop or implement a Brain-on-a-Chip platform must take numerous practical factors into consideration. First, in general, the researcher should decide whether to create an advanced platform in-house or to use a commercial platform (see Ref. 9 for a summary of current commercial Brain-on-a-Chip platforms)—bearing in mind that creating novel tools for brain research requires interdisciplinary expertise in neuroscience, bioengineering, tissue engineering, electrical engineering, materials science, chemistry, cellular biology, and more. Furthermore, before selecting or designing a platform, it is important to identify the properties of the brain that the system must recapitulate. Though one might assume that an “ideal” model should mimic the brain as faithfully as possible along all the criteria outlined in previous sections, e.g., Criteria for a Next Generation Brain-on-a-Chip, in practice, some properties might be more crucial than others for addressing the specific research question at hand (see below for further discussion). The researcher should also take into account various technical and biological considerations, such as the selection of materials, the cells to be used, and the data readouts to be obtained. In what follows, I will elaborate on each of these factors (see Table III for a summary) and will further discuss specific considerations associated with the development of CNS disease models.

**TABLE III.** Practical considerations in developing a Brain-on-a-Chip platform.

*Cell sources*^a^		
Source	Main advantages	Main disadvantage
Cell lines	Not expensive, immortal, accessible, commercially available, easy to culture.	Usually do not properly mimic the native cells' ECM and functionality. Might change genotype and phenotype after many passages.
Primary cells	Best representation of the *in vivo* state.	Expensive, limited number of cells from the same source. Lack of primary human cell sources for CNS.
Embryonic stem cells (ESC)	Good representation of the *in vivo* state. Many protocols for differentiating into the different cells of the CNS. Can create isogenic CNS model.	Might have regulatory issues. Expensive, limited number of cells from the same source.
Induced pluripotent stem cells (iPSC)	Excellent for personalized medicine. Many protocols for differentiating to the different cells of the CNS. Can create isogenic CNS model. Easy to obtain. Excellent for personalized medicine.	Expensive, differentiation protocols might take a long time. The protocols cause the cells to lose many of the epigenetic markers that evolve over the years, and therefore, iPSCs are considered as “young cells,” which might not be appropriate for specific research questions. Risks of mutations.
Mesenchymal stem cells (MSCs)	Easy to obtain. Good candidate for personalized medicine. Not expensive.	Limited number of protocols that can differentiate MSCs into cells from the CNS.
*Readouts (sensors)*^b^		
Cell type	Parameter	Tools
All cells	Secretion of specific protein\molecules	Electrochemical sensors, optical tools
All cells	Morphology	Microscopy and image analysis tools, such as Fiji and Imaris
All cells	Metabolomics	Mass spectrometer
Neurons	Electrical activity	MEA, patch clamp, voltage sensitive dyes or calcium imaging
Neurons	Neurite growth	Microscopy and image analysis tools, such as Fiji and Imaris
Endothelium (and BBB)	Permeability	TEER and fluorescent markers

^a^ The table refers to cells from human sources, but a Brain-on-a-Chip can also integrate cells from animal sources (e.g., rodents). These cells have their own advantages and disadvantages, which are beyond the scope of this Perspective.

^b^ The table present readouts that can be done *in situ* (in the chip). There are also multiple off-chip readouts, such as immunohistochemistry, proteomics, enzyme-linked immunosorbent assay (ELISA), etc.

### Materials and fabrication methods

In most cases, the fabrication methods and the materials used for a Brain-on-a-Chip platform go hand-in-hand, with the former determining the latter (or vice versa). For example, injection molding is limited to thermoplastics, and bioprinting better fit to gels. Accordingly, the fabrication method and materials should be selected carefully, as they can significantly affect various properties of the Brain-on-a-Chip.

In selecting materials, the researchers should consider both the substrate that the cells grow on (e.g., how the material affects the properties of the cells; see discussion of Challenge 1 above) and the materials used for the “chip” containing that substrate. For the latter, material properties that should be taken into account include stiffness, biocompatibility, optical transparency (to allow for imaging the cells), if the material is likely to adsorb solutes that will be perfused in the chip [e.g., polydimethylsiloxane (PDMS) adsorbs hydrophobic compounds], durability, degradation, how easy it is to work with the material, availability, cost, the fabrication methods that are applicable to that material, and the resolution of features that can be produced with the fabrication/material combination (e.g., photolithography can create features at a micrometer resolution, while bioprinting provides significantly lower resolution). In some systems, the substrate and the chip might be made of the same material, but this is not always the case. For example, in a microfluidic platform, the chip can be made of polydimethylsiloxane (PDMS), poly(methyl methacrylate) (PMMA), polycarbonate (PC), or other materials, whereas the cells might grow on glass, gels, or specific polymers. In the case of 3D-printed gels, a 3D structure created by a printer might serve both as the chip and as the material supporting cell growth.

Numerous fabrication methods are currently available, including photolithography, 3D printing, microcontact printing, laser-based patterning, injection molding, and casting. While it is beyond the scope of this Perspective to review all these methods and their associated materials, it is important to note that whereas some methodologies can be easily implemented in most labs (e.g., 3D printing), others require more knowhow and specialized facilities (e.g., photolithography and injection molding). For more information about the materials and fabrication tools used for OoCs, in general, and for Brains-on-a-Chip specifically, see the following reviews.^15,128–133^

### Cell source, culture conditions, and validation

An *in vitro* model is only as good as the cells that compose it. Indeed, many reviews have addressed the advantages and disadvantages of different cell sources in advanced *in vitro* systems^9,65,134–136^ [including primary cells, induced pluripotent stem cells (iPSCs),^137^ mesenchymal stem cells (MSCs),^138^ cell lines, etc.]. Table III summarizes the main cell sources that are available. Though it is beyond the scope of this paper to discuss the various cell sources in detail, it is important to be aware that the selection of cell source and cell type will have a critical effect on the results, costs, and ability to reproduce the results. In particular, different cells can serve different functions. For example, cell lines (which are very easy to work with, affordable and accessible, but lack many of the *in vivo* features) can be useful for optimizing and testing a platform, whereas iPSCs are preferable for “personalized medicine,” as they are easy to obtain, carry one's genetic code, and can be manipulated and differentiated into other cell types.

In addition to selecting an appropriate cell source, it is crucial to verify the cells' functionality *in the platform*, as the platform itself might harm or otherwise alter cells' functionality. For example, brain parenchymal cells—unlike many other tissues, which are exposed to high shear flow (e.g., endothelium and epithelium)—are typically exposed to very low shear stress (approximately 0.01 dyne/cm^2^);^12^ therefore, culturing neurons in a microfluidic channel, under flow, might be harmful for the neurons, if the shear is not well monitored. A recent work by Lu *et al.* further illustrates the importance of validating cell functionality in the platform, in showing that many of the currently used protocols for creating brain pluripotent stem cell (hPSC)-derived brain microvascular endothelial cells (iBMECS) actually create epithelium rather than endothelium.^139^

In general, a variety of approaches can be used for such validation, including the assessment of cellular morphology, protein production/secretion, gene expression, electrophysiological characteristics, and more. Notably, only a few papers discuss the means of assessing cellular functionalities that are specifically required in a Brain-on-a-Chip. For *neurons*, for example, though various biological markers exist (e.g., β-III-tubulin, synaptophysin), one of the best markers is electrical activity, which can be easily measured via calcium imaging, voltage-sensitive dyes, patch clamp, or multi-electrode array (MEA). The *BBB,* too, has several biological markers that can indicate functionality (e.g., ZO-1, connexin 43); moreover, BBB functionality can be assessed using permeability measurements, such as transepithelial/transendothelial electrical resistance (TEER) and fluorescent markers for permeability. The functionality of *oligodendrocytes*, in turn, can be evaluated by the degree of myelination, etc.

Regardless of the specific method used, the characterization of cell functionality is necessary for ensuring that the cells and the conditions under which they are being cultured meet the experiment's criteria—e.g., the cells express all relevant genes and proteins and are at the right level of maturation (not too young or old); Using the appropriate number of passages, and ensuring that it is being executed correctly, the microenvironment is appropriate and contains all necessary nutrients, etc. A setup that fails to demonstrate proper functionality may indicate an underlying problem with one of these features, which, in turn, may significantly affect the results obtained from the platform. Unfortunately, many studies do not include this critical validation step.

### Readouts (sensors)

To be able to obtain functionality measurements in a chip—and to extract any other desired information—it is important to ensure that the platform is equipped with appropriate sensors and other analytical tools. Table III presents a summary of the main tools that are used with Brain-on-a-Chip platforms (see also Refs. 66, and 140–144 for reviews of the various tools available for OoC systems, in general, and see Ref. 35 for specific discussion of Brain-on-a-Chip systems). Some sensors, such as MEA (for electrophysiology) and TEER (for permeability), can be integrated into the chip. In other cases, the chip is integrated into the sensor (e.g., mass spectrometer) or fit to standard analytical tools (e.g., microscope).

### The optimal level of simplicity for a specific application

As noted above, it is not always necessary, or even advantageous, to strive for a model that mimics the maximum possible number of features of the brain. Rather, a model should provide sufficient detail for addressing the focal research questions, while achieving additional practical objectives. The more complex a system is, the harder it is to fabricate, the more expensive it is, and the more challenging it is to maintain. Accordingly, a highly complex and detailed system may be less appropriate for drug screening, for example, in which it is necessary to process thousands of compounds, and high throughput and cost effectiveness may take priority over other features (assuming that the system maintains key functionalities that enable it to provide meaningful information). The answer to the question “How simple is too simple/not simple enough?” is unique to every experiment and platform. In any case, as platform development can take a great deal of time and money, researchers who are just entering the field of Brains-on-a-Chip and do not yet have all the required tools and knowhow might benefit from starting with more simplified models (e.g., Ref. 145) or, alternatively, collaborating with labs that develop such platforms or using commercial ones.

In what follows, I present a basic list of potential applications for Brain-on-a-Chip systems, each of which is likely to be characterized by specific requirements in terms of the features of the platform.

*Drug development*: Pharmaceutical companies have increasingly begun to use OoCs^146^ for expediting the drug development process. When it comes to Brains-on-a-Chip specifically, there is an urgent need for better models of the NVU, in order to assess drugs' capacity to cross the BBB and to affect the brain. Moreover, platforms that enable systemic effects to be monitored—e.g., by linking the Brain-on-a-Chip to other OoCs—are likely to provide a significant utility in drug development applications.

*Personalized medicine:* Recent advances in the iPSC field have opened the door to the creation of personalized models of the NVU.^93,147^ Recently, researchers have begun to integrate iPSCs into OoC systems, providing the capacity to test and screen drugs for specific diseases on an isogenic platform—an approach that has the potential to give rise to more effective treatments.

*Characterization of human physiology:* Human-relevant *in vitro* models, and Brains-on-a-Chip specifically, provide opportunities to investigate the processes that are unique to the human physiology (as opposed to the physiology of other animals) and that cannot be examined *in vivo* in humans. Examples of such applications include the identification of metabolic coupling between the brain endothelium and neurons,^92^ and elucidation of the mechanism for psychomotor retardation associated with mutations in the thyroid hormone transporter MTC-8.^148^

*Experiments in extreme environments:* Researchers are increasingly seeking to understand how human physiology behaves in extreme environments—and particularly in space, given that space travel is becoming more accessible. There is more unknown than known, for example, with regard to how microgravity affects various tissues, including the brain. OoCs (and Brains-on-a-Chip specifically) can be useful in such research, as they provide a means of culturing tissues in “self-maintained” compartments that do not require complex handling or unique expertise for operating the system. Indeed, the National Center for Advancing Translational Sciences has partnered with the International Space Station (ISS) to collaborate on sending OoCs to space, in order to study the effect of microgravity on tissues.^149^

*Modularity and integration with robotic platforms:* The modular and self-maintained nature of OoC platforms enables such systems to serve as links between biotic and abiotic interfaces. In particular, such platforms can enable biological tissues to be integrated with robots. Researchers have recently begun to create biohybrid robots integrating insects and robots,^150^ and in a recent work, researchers used a locust ear to create an “Ear-on-a-Chip” linked to a robot,^151^ or using neuronal system to control flight simulators^73^ and computer software.^74^

### Specific considerations in the development of models of neurological disorders

In recent years, there have been substantial technological leaps in the development of disease models based on Brain-on-a-Chip platforms. These models include cancer,^102^ Parkinson's disease,^152^ Alzheimer's disease,^101^ amyotrophic lateral sclerosis (ALS),^153^ traumatic brain injury (TBI),^154^ fungal infections,^155^ and more. While it is beyond the scope of this perspective to review these models, it is important to note several challenges that are specific to the establishment of CNS disease models in a chip. These challenges include the following: (i) disease mechanism: for most neurological disorders the mechanism is unknown, which makes it very challenging to mimic the disease in an *in vitro* platform; (ii) human relevance: some neurological disorders are unique to humans, and it is challenging to mimic these disorders using non-human tissues; (iii) systemic effects: when mimicking disease it is very important to include the immune system, for identifying the immune response, and to incorporate other relevant organs, such as the liver and kidney, which will metabolize and secrete the drugs given to treat the disease; (iv) readouts: some neurological disorders manifest in cognitive and behavioral changes that are impossible to mimic *in vitro* at this point (Challenge 7); and (v) age: many neurodegenerative diseases appear among elderly individuals; it is highly challenging to work with aging cells or cells with age-related features.

In light of these challenges, and in spite of the advancements of recent years, the development of advanced *in vitro* models of neurological disorders remains an acute problem.

### Summary of considerations in the development and implementation of a Brain-on-a-Chip

Summing up the discussion above, it is clear that there is no “one Brain-on-a-Chip fits all” model; rather, different applications require different platforms, which may entail unique sets of practical considerations. Accordingly, the researcher who aims to develop and use such a platform should follow these basic steps:

(1) Clearly define the problem you are trying to solve.
(2) Identify the most significant parameters that can affect the results (e.g., whether it is imperative to include flow, a 3D structure, multiple cell types, etc.)
(3) See which platforms are currently available that will enable you to incorporate the parameters you are interested in (taking into account the parameters mentioned above, e.g., readouts, cells, and materials). If the platforms are not commercially available, you will need to design, build, and test the platform.
(4) Is it a Brain-on-a-Chip? Once the platform is planned, the researcher can determine whether it qualifies as a Brain-on-a-Chip by calculating a score for the system based on the criteria in Table I. For example, if the platform is 3D (1), includes flow (1), and has multiple cell types (1), the total score is 3, which is greater than 2—which means that the system qualifies as a Brain-on-a-Chip (according to current criteria).

## FUTURE DIRECTIONS

Many of the fundamental challenges of *in vitro* modeling of the brain are attributable to the simple fact that the brain is highly complex (Figs. 1 and 2), and we possess insufficient knowledge on how it works, or even on how it is composed and structured. Clearly, it is difficult to mimic such a system, even with the most advanced *in vitro* technology. Thus, current Brain-on-a-Chip systems mainly focus on mimicking the basic biological functions and interactions of the cells that compose the CNS. Yet, as China,^156^ the US, and Europe are currently investing major efforts in better characterizing the brain and understanding how it functions (e.g., BRAIN and HBP initiatives for $1 billion USD and 1 billion Euros, respectively,^157^ as well as China's Brain Project^158^), it seems likely that new insights will emerge that will contribute toward the advancement of Brain-on-a-Chip models. For example, future systems may be less restricted to a focus on basic biological processes (e.g., neuronal electrical activity) and may have the capacity to provide measurements of other forms of functionality, such as network activity and plasticity, and perhaps even advanced functionalities, such as cognition.

Such developments are likely to be facilitated by the increasingly powerful computational tools at our disposal, such as machine-based learning, deep learning, and artificial intelligence, especially in the field of neuroscience.^159,160^ These tools can assist in identifying electrophysiological signals and translating them into voice, words, and other physiological outputs.^161^ It seems plausible that, eventually, these capabilities might be incorporated into *in vitro* OoC platforms, to better represent *in vivo* activity.

On a more technical level, the discussion above highlights the fact that, though current Brain-on-a-Chip systems are capable of tackling some of the challenges associated with *in vitro* CNS modeling (Table I), none addresses all of them. It seems likely that future Brain-on-a-Chip systems may have this capacity, through the use of advanced biomaterials that better recapitulate the *in vivo* microenvironment as well as integration of multiple cell cultures representing different brain subunits, brain regions, and organs. Tools, such as iPSCs and gene-editing capabilities, may further enable Brain-on-a-Chip technologies to be combined with personalized medicine approaches—providing powerful platforms for drug screening and disease modeling. One possibility is that the diverse approaches to the Brain-on-a-Chip will converge to a standard model, e.g., a system of integrated units comprising a BBB, microvasculature, and a neuronal compartment, integrated with sensors, for real-time assessment of BBB permeability and neuronal electrical activity.

## Data Availability

Data sharing is not applicable to this article as no new data were created or analyzed in this study.
